# Delayed endovascular revascularization of renal artery bridging stent occlusion after complex endovascular aortic repair

**DOI:** 10.1186/s42155-026-00696-6

**Published:** 2026-05-16

**Authors:** Stevo Duvnjak, Kim Kargaard Bredahl, Antonia Rinaldi, Timothy Andrew Resch

**Affiliations:** 1 Department of Vascular Surgery, Heart Center, Copenhagen University Hospital - Rigshospitalet, Copenhagen, Denmark; 2 Department of Clinical Medicine, University of Copenhagen, Copenhagen, Denmark

**Keywords:** Fenestrated endovascular aortic repair (FEVAR), Branched endovascular aortic repair (BEVAR), Renal ischemia, Stent, Thrombosis

## Abstract

**Background and purpose:**

Recanalisation of renal artery bridging stent graft thrombosis in patients with delayed ischemia lasting more than 24 h could potentially save some residual renal function. This study evaluates the technical success and short-term clinical outcomes of delayed recanalisation of bilateral or single-functioning kidneys with renal artery occlusion after endovascular repair of complex aortic aneurysms.

**Materials and methods:**

We retrospectively analysed 11 patients treated between October 2019 and November 2024 who developed occlusion of a single functioning kidney or bilateral renal stent-graft thrombosis. Technical success was defined as recanalisation of at least one occluded renal bridging stent with restoration of blood flow to the kidney.. Clinical success was, defined by the improvement or stabilisation of residual renal function (eGFR) and delaying the need for dialysis.

**Results:**

Delayed endovascular repair was performed for fifteen renal artery stent-graft thromboses in eleven patients.. Mean age was 68.7 ± 5 years; nine patients (81%) were male. Six patients (54%) had contained aortic rupture or aneurysms > 8 cm and were treated acutely/subacutely with off-the-shelf stent grafts; four (36%) received custom-made devices, and one a fenestrated cuff. Main symptoms included anuria (81%), nausea, diarrhoea, and flank pain (100%). Time from symptom onset to treatment ranged 24–96 h (mean 27.2 h), and mean time from index procedure to thrombosis was 10.4 months. Clinical success was achieved in 72% of cases. Nine patients required dialysis post-intervention; six were temporary, and three permanent. Perioperative complications occurred in 2/11 patients. In 55%, the cause of occlusion was undetermined. Median follow-up was 18.5 months (IQR 0–33).

**Conclusion:**

Delayed renal stent graft recanalisation is safe and effective, preserving renal function and avoiding dialysis in single-functioning or bilateral renal artery occlusions. Recanalisation should be considered aggressively when renal perfusion remains, regardless of occlusion duration.

## Introduction and purpose

Complex aortic endovascular procedures, including fenestrated (FEVAR) and branched repair (BEVAR), are typically the preferred treatment for thoracoabdominal aortic disease due to lower morbidity and mortality rates compared to open surgery [1].

One potential complication is target-vessel stenosis and thrombosis, particularly in renal arteries supplied by branches [2]. According to the literature, the incidence of renal bridging stent thrombosis is approximately 9% during medium-term follow-up and increases to 13% in BEVAR cases [2, 3]. Renal stent graft thrombosis is especially concerning in patients with bilateral disease or those with a single-functioning kidney. In those patients, loss of both or only one functional kidney results in dialysis unless fast clinical recognition and recanalisation are performed to try to save any kidney function.

In previous studies, renal ischemia lasting more than six hours was considered beyond salvage and attempts at renal reperfusion might be harmful [4]. Consequently, patients with prolonged renal ischemia are often considered ineligible for interventions aimed at restoring renal function. In some cases, successful outcomes following delayed (> 6 h) treatment of renal stent graft thrombosis after FEVAR/BEVAR may be due to pre-formed collateral vessels [5, 6]. However, there is still no consensus on the optimal timing or method of treatment, largely due to limited data and small patient cohorts.

The primary aim of this study is to evaluate and analyse the technical success and short-term clinical outcome of delayed recanalisation of either bilateral renal artery occlusion or only one functional kidney renal artery occlusion after endovascular repair of complex aortic aneurysms.

## Materials and methods

Delayed endovascular revascularisation of 15 renal artery bridging stent graft thromboses in 11 patients was performed. All patients presented with imaging-confirmed thrombosis or occlusion of the renal artery bridging stent graft.

Technical success was defined as successful recanalisation of the occluded renal bridging stent graft with restoration of blood flow in the kidney, even if some residual thrombus remained visible in the segmental arteries. The unsuccessful recanalisation was defined as the failure to restore blood flow to the kidney.. Clinical success was defined as the patient being free from dialysis (for patients who had required dialysis) and the return of renal function to baseline level.

The need for temporary and permanent dialysis was recorded. Renal function was assessed at baseline before the index intervention, during admission when renal artery occlusion occurred, and throughout recovery and follow-up using the changes in estimated glomerular filtration rate (eGFR) and the difference in creatinine levels. Renal function impairment was classified according to the chronic kidney disease (CKD) classifications [7].

Data on the type of aortic stent graft and the type of bridging stent used for primary treatment, onset of symptoms and time to intervention, access route, type of intervention, technical success, complications, mortality, secondary interventions, and maximum follow-up duration were recorded. The surveillance protocol consisted of a computed tomography scan (CT) with or without contrast, based on residual renal function assessment at 3 months post-recanalisation, followed by annual imaging thereafter.

Briefly, the recanalisation technique consisted of a 7-Fr TourGuide steerable sheath (Medtronic, Minneapolis, USA) being navigated to the renal branch. A 0.035-inch, 260-cm hydrophilic guidewire and a 5-Fr support catheter were used for recanalisation. The guidewire was then exchanged for a 260-cm Rosen stiff guidewire (Cook Medical, Bloomington, IN, USA), and a new stent graft was deployed over the stiff guidewire. Finally, post-dilation was performed using a balloon with a diameter 1 mm smaller than the deployed stent graft. In two patients with brachial access, a long 90-cm 6-Fr sheath was used to engage the renal branch, and the same technique was used afterwards. Perioperative complications occurred in two patients. In two patients with brachial access, a long 90-cm 6-Fr sheath was used to engage the renal branch, and the same technique was used afterwards.

Ethical approval was waived by the regional ethics committee. 

### Statistical analysis

Normally distributed variables are presented as mean and range and non-normally distributed variables as median and interquartile range (IQR). Categorical variables are presented as numbers and percentages. Paired Student’s t-test was used for statistical analysis, and a *p*-value < 0.05 was considered statistically significant.

## Results

Eleven patients underwent renal artery endovascular recanalisation of 15 bridging renal artery stent graft thrombosis and are included in a study. Four patients (36%) had a single functioning kidney due to previous asymptomatic renal stent graft thrombosis in the contralateral artery. Four patients (36%) had bilateral renal stent graft thrombosis or occlusion. Three patients (27%) had a solitary kidney after prior nephrectomy at the time of the primary repair. Consequently, the entire group of patients without revascularisation attempts will be dialysis patients. In all, 363 patients with complex aortic aneurysms were treated with FEVAR/BEVAR at a tertiary referral center between October 2019 and November 2024. None of the study patients was on dialysis preoperatively, and they had either normal renal function or mild renal impairment according to the CKD classification. Four patients (36%) had normal > 90 mL/min/1.73 m^2^ eGFR, and seven patients (64%) had mildly impaired renal function eGFR < 90; > 40 mL/min/1.73 m^2^ (CKD: 1–3). Demographic baseline data and indications for treatment are listed in Table 1. Generally, in our practice, during the first 3 months after F/BEVAR, all patients are on dual antiplatelet therapy (acetylsalicylic acid 75 mg plus clopidogrel 75 mg), followed by single antiplatelet therapy thereafter. Six patients (54%) presented with either a contained aortic rupture or an aneurysm size > 8 cm and were treated acute/subacute using an off-the-shelf COOK T-branch device (COOK Medical, Bjaevreskov, Denmark); four patients (36%) received custom-made devices with inner branches (Artivion, Hechingen, Germany); one patient (9%) was treated with a custom-made devices fenestrated cuff (COOK Medical Inc, Bjaevreskov, Denmark) due to type 1 endoleak after previous EVAR. Indications for treatment, type of stent graft, and periprocedural characteristics are listed in Table 2.

**Table 1:** Demographic characteristics, baseline eGFR function and treatment type

Value	Number (%)
Age Male	68.7 ± 5 9 (81%)
Hearth comorbidities (coronary ischemia/congestive heart diseases	7 (63%)
COPD-chronic obstructive pulmonary disease	4 (36%)
Hypertension arterials	11 (100%)
Diabetes mellitus	3 (27%)
eGFR > 90 ml/min	4 (36%)
eGFR < 90, > 40 ml/min	7 (63%)
eGFR < 40 ml/min	0
Emergency FEVAR/BEVAR	6 (54%)
Elective FEVAR/BEVAR	5 (46%)

**Table 2:** Aortic pathology, aortic and renal stent grafts, recanalisation strategy and periprocedural complications

Pt	Aortic Disease	Aortic Stent Graft	Renal Stent Graft (Index)	Renal Stent Graft (Recanalization)	Uni/bilateralt	Intervention	Periprocedural Complications
1	Type I endoleak -previous EVAR	Fenestrated COOK Medical aortic cuff	Advanta 6 × 22 mm	Advanta 6 × 22 mm + self-expandable stent 7 × 20 mm	Unilateral	Local thrombolysis + relining	None
2	Juxtarenal AAA	T-Branch COOK Medical	VBX 6 × 79 mm	BeGraft 6 × 57 mm	Bilateral	Aspiration thrombectomy + relining	Access-site bleeding requiring open surgery
3	TAAA type I	T-Branch COOK Medical	BeGraft 6 × 57 mm	Viabahn 6 × 50 mm	Unilateral	Local thrombolysis + relining	None
4	TAAA type II	T-Branch COOK Medical	Covera 8 × 80 mm (right previously occluded)	BeGraft 7 × 57 mm + BeGraft 6 × 38 mm + self-expandable stent 7 × 30 mm	Unilateral	Aspiration thrombectomy (Penumbra) + relining	None
5	Chronic type B dissection with TAAA	T-Branch COOK Medical	VBX 6 × 59 mm + Viabahn 6 × 50 mm	Viabahn 6 × 50 mm + self-expandable stent 7 × 30 mm	Unilateral	Aspiration thrombectomy + relining	None
6	TAAA type IV	T-Branch COOK Medical	BeGraft 8 × 57 mm + Viabahn 7 × 50 mm	Viabahn 6 × 50 mm (left unsuccessful)	Bilateral (left unsuccessful)	Aspiration thrombectomy + relining	Bleeding after recanalization
7	TAAA type IV	T-Branch COOK Medical	VBX 6 × 59 mm (prior left nephrectomy)	BeGraft 6 × 57 mm + self-expandable stent 6 × 20 mm	Unilateral	Aspiration thrombectomy + relining	None
8	TAAA type III	E-nside (Artivion)	VBX 7 × 79 mm	VBX 6 × 79 mm	Bilateral (right unsuccessful)	Aspiration thrombectomy + relining	None
9	TAAA type III	E-nside (Artivion)	Viabahn 6 × 100 mm + Advanta 6 × 22 mm	Viabahn 6 × 50 mm + BeGraft 6 × 22 mm	Bilateral (left unsuccessful)	Aspiration thrombectomy + relining	None
10	TAAA type III	E-nside (Artivion)	VBX 8 × 59 mm + BeGraft 7 × 57 mm	BeGraft 7 × 57 mm + self-expandable stent 9 × 20 mm	Unilateral	Local thrombolysis	None
11	TAAA type IV	E-nside (Artivion)	Viabahn 6 × 100 mm + BeGraft 6 × 57 mm	VBX 6 × 58 mm	Unilateral	Aspiration thrombectomy + relining	None

Advanta V12 (Getinge/Covidien, Minneapolis, MN, USA)

VBX (W.L. Gore & Associates, Flagstaff, AZ, USA)

BeGraft (Bentley InnoMed GmbH, Hechingen, Germany)

Viabahn (W.L. Gore & Associates, Flagstaff, AZ, USA)

Covera (Bard Peripheral Vascular, Tempe, AZ, USA)

EvaFlex (Medtronic, Minneapolis, MN, USA)

Pulsar stent–Biotronik, Bülach, Switzerland

**Fig. 1:**
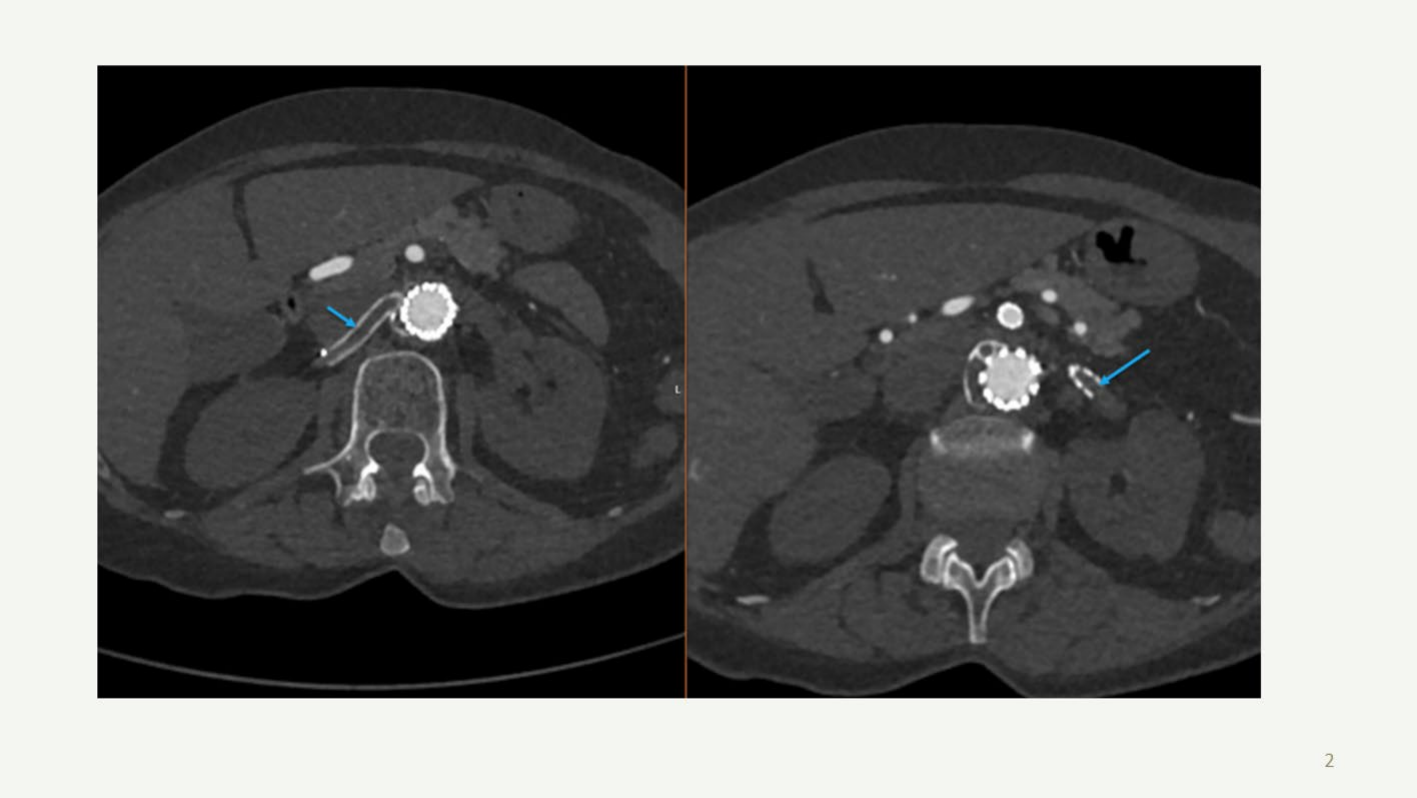
Contrast-enhanced computed tomography (CT) with axial reconstruction shows occluded bilateral renal stent grafts (arrow) after BEVAR in a 73-year-old patient with a contained aortic aneurysm rupture. The patient was transferred to our hospital > 24 h after the onset of nausea, anuria, and lumbar pain. Baseline eGFR was 75 mL/min at the index BEVAR procedure eleven months earlier. After renal stent graft thrombosis, eGFR decreased to 3 mL/min

**Fig. 2:**
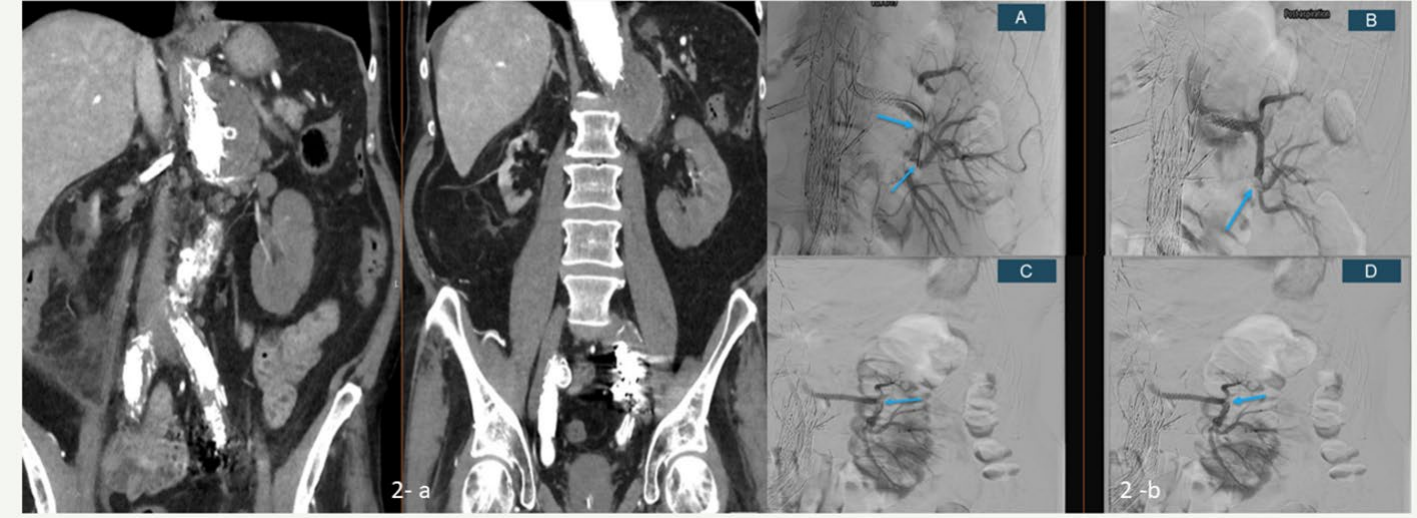
A 65-year-old patient with a thoracoabdominal aortic aneurysm treated with BEVAR. Baseline eGFR was > 90 mL/min. Two months after BEVAR, the patient was admitted to another hospital with anuria and back pain. The patient was transferred to our hospital more than 24 h after symptom onset with an eGFR of 9 ml/min. Contrast-enhanced computed tomography with coronal reconstruction showed occluded bilateral renal stent grafts and atrophy of the right kidney (**a**). **b** Recanalisation of the left renal stent graft and relining with a Viabahn stent graft, with a successful outcome despite some residual thrombus (**A**-**D**-arrows). The patient is not on dialysis, and the eGFR is 37 mL/min

**Fig. 3:**
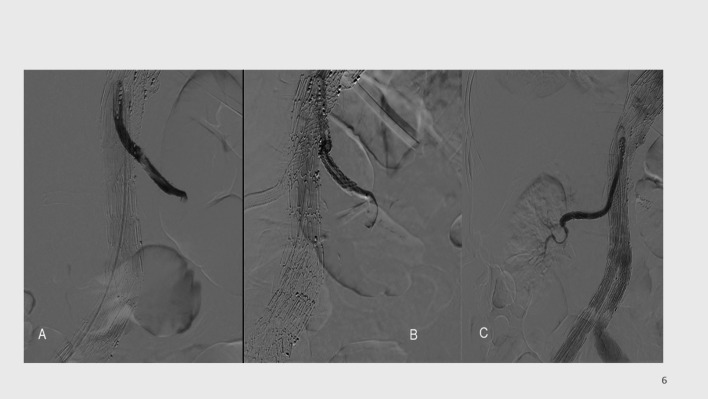
A 76-year-old patient with a thoracoabdominal aortic aneurysm was treated with T-branch off-the-shelf stent graft. Baseline eGFR was 89 mL/min. Three months after BEVAR, the patient was admitted to our hospital four days after the onset of anuria. Bilateral stent graft thrombosis was confirmed on CT, and recanalisation of both renal arteries was attempted. The left renal artery could not be recanalised, and there was no flow in the segmental renal arteries (**A**, **B**). The right renal artery was successfully recanalised, with restoration of flow in the main renal artery and two segmental arteries; however (**C**), there was no clinical improvement, and the patient remains on permanent dialysis

**Fig. 4:**
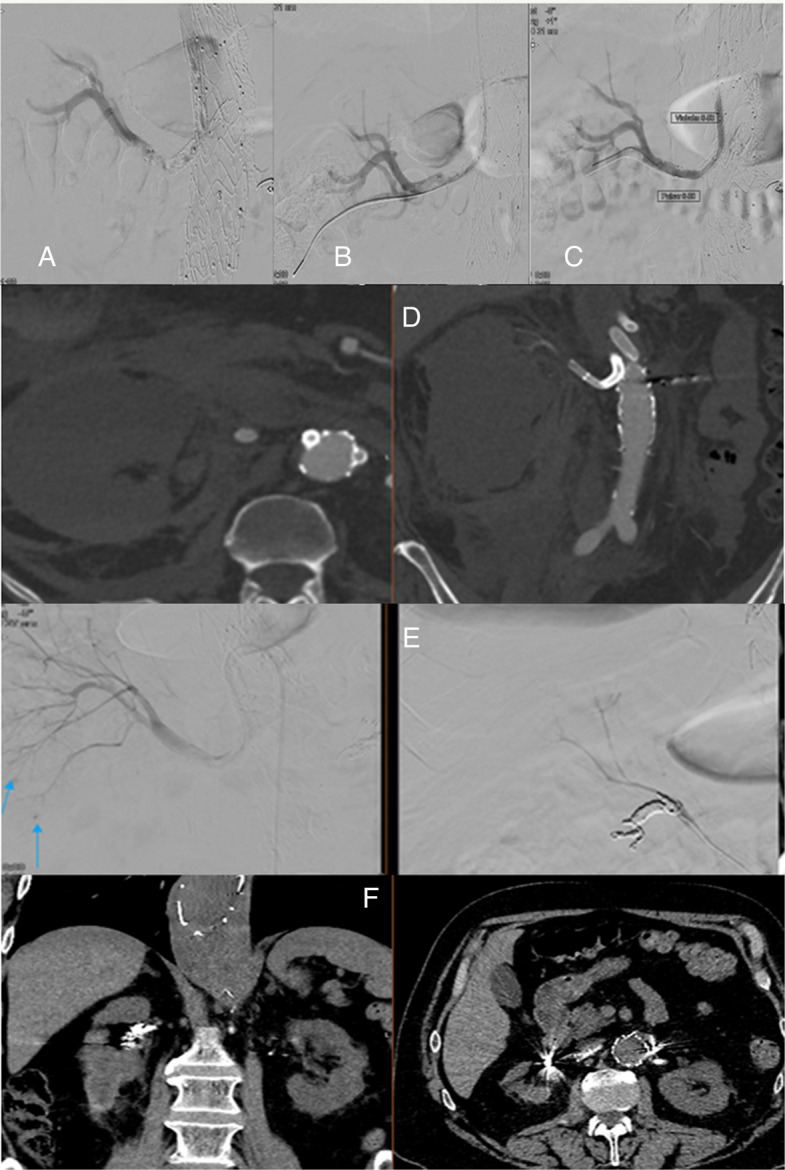
The same patient shown in Fig. 1 became hemodynamically unstable a few hours after the procedure. **A** Right renal DSA after recanalisation shows residual thrombus within the stent and no flow distal to the segmental arteries. **B** The tip of the catheter is in a distal-pole segmental branch. **C** After withdrawal of the catheter or a guidewire, a significant narrowing is seen at the proximal part of the renal artery stent graft with no evidence of blood flow distal to the segmental arteries. **D** CT shows renal and perirenal hematoma with no active contrast extravasation. There is no blood flow in the intra-renal arterial branches. **E** Angiography was performed, and embolisation of the lower pole of the right kidney was performed with 3 mm and 5 mm microcoils. The patient required temporary dialysis but eventually recovered. At present, the patient is off dialysis, with severely impaired renal function (eGFR 17 mL/min) and preserved diuresis. **F** A follow-up non-contrast CT scan demonstrated a reduction in renal parenchymal volume

The mean age was 68.7 ± 5 years, and nine patients (81%) were male. Contrast-enhanced CT scans confirmed renal stent graft thrombosis (Fig. 1) in all cases. The mean time from index FEVAR/BEVAR to renal stent graft thrombosis was 10.4 months (range 2–48 months). In all patients, no issues with the renal graft were detected on the last available follow-up CT imaging before thrombosis occurred. The main symptoms included anuria in nine patients (81%), nausea, diarrhoea, and abdominal flank pain in 11 patients (100%). In all patients, the time from symptom onset to imaging confirmation of renal ischemia was more than 24 h. The delay from diagnosis to initiation of treatment ranged from 24 to 96 h, with a mean of 27.2 h. Restoration of blood flow to at least one kidney after recanalisation was achieved in all patients (see an example in Fig. 2). In four patients with bilateral bridging stent graft occlusion, recanalisation of both renal stent grafts was attempted; however, only one renal bridging stent graft was recanalised but without clinical renal function recovery in three patients (see an example in Fig. 3). Perioperative complications occurred in two patients. These included bleeding from a renal segmental branch during recanalisation caused by guidewire perforation, which was not detected during the intervention or on control angiography. A control CT scan performed a few hours after the procedure revealed a retroperitoneal hematoma on the right side. Embolisation with microcoils was subsequently performed, successfully achieving hemostasis (Fig. 4). Another patient developed bleeding and hematoma at the brachial access site, which required open surgical repair.

**Fig. 5:**
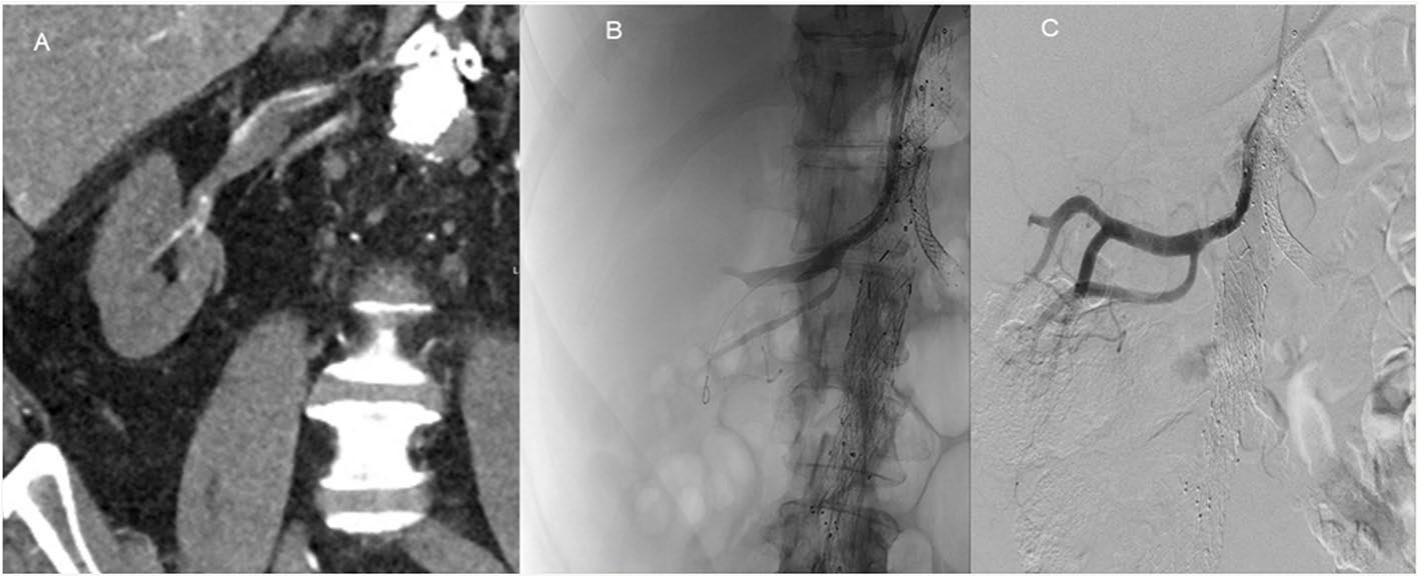
A 68-year-old patient was treated with BEVAR due to a contained rupture of a type IV aortic aneurysm. Baseline eGFR was 88 mL/min. Four months later, the patient was admitted with anuria and back pain lasting more than 24 h. Contrast-enhanced CT showed bilateral renal artery thrombosis. The left renal artery could not be recanalised. The right renal artery was treated with local thrombolysis with a good result (**A-C**), and the patient is not on dialysis, with an eGFR of 40 mL/min

Seven patients (63%) underwent relining with a new stent graft combined with simple manual aspiration thrombectomy. Two patients were treated with two days of thrombolysis using alteplase at 0.5 mg/hour, followed by relining. One patient underwent endovascular thrombectomy with the Penumbra Indigo (Alameda, CA, USA) device followed by relining. A single patient had a satisfactory outcome after one day of thrombolysis alone, with a good clinical outcome and without the need for dialysis (Fig. 5). Femoral access was used in nine patients (82%) and brachial access in two (18%). There was no perioperative mortality.

Nine patients required dialysis after intervention; in six of these cases, dialysis was temporary, while three patients (33%) required permanent dialysis. In those three patients, there was no recovery of any residual renal function despite at least one technically successful open renal branch, and therefore, permanent dialysis was necessary.

Mean value of eGFR before primary operation was 76 mL/min/1.73 m^2^ (range:51–90). Mean eGFR was 8.9 mL/min/1.73 m^2^ (range:8–40) at the acute event with renal thrombosis, and mean eGFR after recanalisation in follow-up was 27.8 mL/min/1.73 m^2^ (range 5- 49). Mean creatinine before primary operation was 87.5 µmol/L (range:53–161), creatinine value at renal graft event thrombosis was 627 µmol/L (range: 159–1530), and in follow-up, the mean value was 250 µmol/L(range 94–612).

Significant deterioration of renal function in all cases occurred and remained significantly reduced compared with baseline renal function in the follow-up period, calculated by eGFR (*p* < 0.001) and creatinine course (*p* < 0.001).

The cause of renal stent graft occlusion could not be determined in 55% of patients. In 45% (*n* = 5), occlusion was attributed to mechanical stent compression (*n* = 4) or discontinuation of dual antiplatelet therapy due to scheduled biopsy (patient = 1).

Overall, two patients died during the follow-up period. One patient died within 30 days due to multiple organ failure, and another died 2 months post-recanalisation from intracerebral bleeding of unknown cause. The patient was discharged after recanalisation and was prescribed dual-antiplatelet therapy. The cause of the intracerebral bleeding could not be established with certainty.

The median follow-up after recanalisation was 18.5 months (IQR, 0–33). No secondary interventions on renal stent grafts were required during follow-up, and no mortality was obsserved.

## Discussion

We present our experience with recanalisation of renal artery bridging stent graft thrombosis after B/FEVAR in patients with either delayed ischemia lasting more than 24 h in a single functioning kidney or bilateral stent graft thrombosis. Without an attempt at revascularisation, all of these patients would likely have required dialysis, with the associated limitations, complications, and costs. In 8/11 patients (72%), some residual renal function recovered, allowing life without dialysis. 10/11 patients presenting had BEVAR, and 6/11 were originally treated in an urgent setting.

Improvement in quality of life is a key outcome for these patients, and with a relatively simple and fast endovascular procedure, renal function can be restored to a sufficient level to permit dialysis-free living. However, significant impairment in renal function was observed in nearly all patients, and a future episode of renal thrombosis may likely result in permanent dialysis dependency.

The published literature reports ischemia times ranging from a few hours to more than 24 h, with most cases achieving high technical success rates and low complication rates. Collateral flow from lumbar and other vessels is thought to provide some kidney perfusion in cases of main renal artery thrombosis [5, 6]. From a clinical perspective, the appearance of contrast in either the renal parenchyma or in the renal artery distal to the occlusion can be used as a sign of renal viability and thus serve as a basis for potential renal salvage.

There is currently no consensus on the optimal technique for recanalisation to rescue renal function. In the literature, various treatments have been reported, ranging from relining and aspiration thrombectomy to thrombolysis [2, 3, 5–7]. A combination of mechanical thrombectomy and relining likely represents the fastest and most reliable approach. With the availability of steerable sheaths, transfemoral access is now almost always feasible, reducing the risk of stroke associated with upper limb access. To the best of the authors’ knowledge, there is no description of an open surgical treatment option in such cases, which emphasises the potential for renal salvage using a minimally invasive approach. In our series, 2/11 patients experienced perioperative complications. It is very important to maintain control over the guidewire when exchanging different devices, as this can help prevent bleeding complications and further impairment of already damaged renal function. The The The availability of steerable sheaths facilitates catheterisation of the renal artery from a transfemoral approach following B/FEVAR and can reduce the need for upper arm approaches..In nearly half of the cases, the exact cause of bridging stent graft thrombosis could not be clearly identified, consistent with other reports. On the previously available control CT, no issues with the bridging stent graft were detected as well. The most cited cause remains mechanical compression [5, 6]. A recent publication reported a patency loss of 17% at 24 months and up to 41% at 48 months in cases involving inner-branched stent grafts [8]. In the current report, most cases were either after emergent cases using an off-the-shelf BEVAR or after CMD devices with inner branches. Only one case was seen after FEVAR, despite this being the vastly predominant procedure at our center during the time. Longer bridging stents and motion during respiration are known risk factors for bridging stent graft thrombosis.. There is no universal follow-up protocol for these patients; instead, individual centers typically develop their own strategies. In our department, follow-up imaging is performed at 3 months post-op and then annually. It is difficult to argue that more frequent follow-up would reliably detect early thrombosis before complete occlusion, so a universal prevention strategy remains elusive.

Dual antiplatelet therapy (acetylsalicylic acid 75 mg plus clopidogrel 75 mg) is usually administered for the first 3 months after F/BEVAR, unless anticoagulant therapy is prescribed due to pre-existing heart disease. In the relevant literature, a similar strategy is generally used, with dual antiplatelet therapy recommended for at least 1 month and up to 6 months after the intervention. There is no strong evidence that prolonged dual antiplatelet therapy prevents eventual stent graft thrombosis [9, 10]. Furthermore, the risk of bleeding is not negligible when a dual-antiplatelet regimen is used over an extended period.

Cone-beam CT (CBCT) is an important part of these procedures and is strongly recommended. In our series, we did not perform any additional interventions based on CBCT findings, although the literature reports that up to 17% of clinically significant structural issues can be detected on CBCT after FEVAR [11]. We performed a CBCT without contrast to assess for any possible stent compression; therefore, no additional contrast was administered. The inner diameter of the aorta at the visceral segment is a critical factor; when the diameter is < 24 mm, the risk of bridging stent graft compression increases. However, in acute situations, real-world clinical judgment often necessitates proceeding with less-than-ideal anatomy to save lives.

Technical success rates are high both in the literature and in our experience. Stent graft thrombosis can occur both early and late after FEVAR/BEVAR, necessitating lifelong surveillance and a low threshold for secondary intervention if any sign of compression is noted on follow-up CT imaging.

In our cohort, bilateral renal bridging stent graft thrombosis occurred in 36% of patients. However, emergency treatment was indicated in only half of these cases. This raises questions about the increasing incidence of such complications in acute settings and the dilemmas regarding their management, including potential violation of the device’s instructions for use. The small number of patients in our study prevents definitive conclusions, but it highlights the need for further discussion, data accumulation, and acknowledgement of the study’s limitations.

## Conclusion

Renal bridging stent graft thrombosis is not infrequent, and treatment is challenging. It seems to be more frequent after BEVAR use, particularly in the acute setting. Delayed renal stent graft recanalisation is a safe and efficient intervention that can preserve residual renal function and avoid dialysis in a single-functioning kidney or in bilateral stent graft thrombosis. The occlusion time alone should not be the deciding factor in pursuing recanalisation. Instead, we advocate for a more aggressive approach in cases of renal stent graft thrombosis, particularly when signs of kidney perfusion remain.

## Data Availability

Yes.
